# Conceptual Framework of Upper Cross Syndrome: A Delphi Study

**DOI:** 10.7759/cureus.67873

**Published:** 2024-08-26

**Authors:** Sharmila Chaudhuri, Meena Gupta, Vandana Phadke, Jasmine Kaur Chawla

**Affiliations:** 1 Physiotherapy, Amity Institute of Health Allied Science, Noida, IND; 2 Clinical Research, Indian Spinal Injury Centre, New Delhi, IND; 3 Physiotherapy, School of Allied Health Sciences, Manav Rachna International Institute of Research and Studies, Faridabad, IND

**Keywords:** conceptual framework, muscular imbalance, postural dysfunction, delphi study, upper cross syndrome

## Abstract

Background: Upper cross syndrome (UCS) is one of the most common postural dysfunctions due to prolonged flexed attitude. Good posture plays an important role in maintaining musculoskeletal balance and protecting it from further injury. Conducting research on the conceptual framework will help clinicians to identify and plan treatment strategies for the correction of this syndrome and prevent secondary complications. Thus, the aim of this study was to develop a conceptual framework for UCS.

Methods and material: The Delphi method was used to conduct the study and develop a conceptual framework. A total of 30 multidisciplinary experts participated in the study, and a list of 41 items was finalized after an extensive literature review. A cover letter along with all the items was mailed to the experts to obtain their input. Three rounds of Delphi were conducted until a consensus was reached. The following parameters were used to determine consensus: moderate Kendall's coefficient of concordance (Kendall's W), agreement greater than 51.0%, interquartile range (IQR) below 1.5, and standard deviation (SD) below 1.

Results: By the end of the third round, 37 items were finalized. The conceptual framework consisted of four items in postural alignment, eight items in muscular imbalance, 20 items in functional limitation, and five items in the psychosocial domain.

Conclusion: We successfully developed a conceptual framework for UCS. Four domains, including postural alignment, muscular imbalance, functional limitation, and psychosocial, were identified. This will lead to a deeper comprehension of UCS, which will facilitate its early detection and treatment. The multidimensional approach of the study will provide a better scope for the clinicians to educate the patient about good posture, which not only will impact physical performance but also improve quality of life. The development of this framework will help to prevent, monitor the progress, and correct UCS.

## Introduction

With the advancement of technology, humans have become more dependent on mobiles and laptops. Maintaining a flexed posture for a long duration of time can lead to upper cross syndrome (UCS). This syndrome is characterized by forward head posture, increased kyphotic angle, and a rounded shoulder [1]. In this syndrome, there is overactivation of the upper trapezius, pectorals, and levator scapula and underactivation of the serratus anterior, infraspinatus, middle, and lower trapezius muscles [2]. These alterations can lead to multiple problems in the head, neck, and shoulder resulting in headache, neck and back pain, chest discomfort, upper arm neuropathy, and gastroesophageal symptoms [3,4].

Musculoskeletal problems can lead to loss of productivity, treatment costs, functional disability, and absenteeism from work [5]. The current literature focuses more on muscular imbalance, which leads to an emphasis on treatment primarily on stretching of tight muscles and strengthening of weak muscles, neglecting motor control deficits, and sustained poor posture [6]. A multimodal approach to the interaction between biomechanical, individual, and psychosocial factors will provide a framework for integrating clinical findings with the diagnosis.

Earlier, in a few studies, a comprehensive approach had been planned for treatment as UCS could be due to sensorimotor dysfunction [6,7]. A deep knowledge of factors associated with UCS will help clinicians identify and plan preventive strategies to deal with this syndrome. There is a lack of studies on proper assessment, correction, and prevention of secondary complications that arise due to this syndrome. Thus, the objective of the present study was to develop a conceptual framework for UCS. This model will help to highlight the multifactorial nature of UCS and understand the complex nature of the interactions between occurrence and response. This model can also be considered as a tool for further research planning and study interpretation.

## Materials and methods

This was an online study conducted by the Delphi method between March and November 2023 at the Department of Physiotherapy, Amity Institute of Health Science, Amity University, Uttar Pradesh, India. The study was approved by the Institutional Ethical Committee of Amity University (approval number: AUUP/IEC/JUN/2022/6).

Delphi is a method to collect information from the experts and to establish consensus on a given topic [8]. It is a structured method in which controlled feedback is taken from the experts and converted into a group consensus [8,9]. It gives inputs on a research problem, making the understanding of the problem better [10]. There is no standard criteria for selection of experts, and the number of experts can vary from 10 to 1000 [11]. However, 30-50 panelists are optimum for conducting Delphi studies. The strength of the Delphi study is the anonymity of the panelists. This helps to reduce dominance and group conformity [11,12]. Delphi is an iterative process that maintains the anonymity of the participants without being biased due to face-to-face interactions, giving more accurate and valid judgment [13].

The criteria for selecting experts were on the basis of experience and knowledge. A heterogeneous panel is considered appropriate in a broader situation; therefore, multidisciplinary experts having more than five years of experience with a special interest in posture and spine or publications were invited to participate in the study. Purposive sampling was used to select the participants. Multidisciplinary experts from various fields were chosen, and invitations were sent to 40 experts, but only 30 of them agreed to participate in the study. A total of 30 multidisciplinary experts were included in the online Delphi study to develop a conceptual framework for UCS. The expert panel consisted of orthopaedic surgeons, physiotherapists, physicians, occupational therapists, and ergonomists from different government, private hospitals, and clinical settings.

An extensive literature review was done using databases like PubMed, EBESCO (Elton B. Stephens Company), MEDLINE (Medical Literature Analysis and Retrieval System Online), and Cochrane using the terms “upper cross syndrome", "postural dysfunction", "forward head posture", "rounded shoulder", and "neck pain". All the studies published between 2000 and 2023 were included, irrespective of their study design. Only full-text articles in English were considered. Based on the clinical relevance, four domains were identified, and all the items were listed under those four domains.

We conducted the online Delphi study by sending a cover letter and a list of items to the experts by email. All the instructions were given to the panelists, and demographic data was obtained. The study had multiple rounds. Each round was conducted for two weeks. A five-point Likert scale was used to score the items (strongly disagree-1, disagree-2, neutral-3, agree-4, and strongly agree-5). The study included multiple rounds. A total duration of two weeks was given to each participant. The gap between the first and second rounds was two weeks.

In the first round, a cover letter along with the list of the items was sent to the experts, and all of them were asked to give feedback. Items from the literature review and all the inputs from the experts were clubbed together to form a pool of 41 items.

In the second round, the experts were asked to rate all the questions on a five-point Likert scale. The judgment was based on the percentage of agreement (which should be at least 51%), interquartile ratio (IQR) (less than 1.5), and standard deviation (SD) (less than 1) [14].

After two weeks, the third round was conducted. The group responses with their own individual responses were sent back to the experts. The experts had the chance to re-evaluate their choices.

Data analysis

All the data were transferred to IBM SPSS Statistics for Windows, Version 21.0 (Released 2012; IBM Corp., Armonk, New York, United States). The percentage of agreement for each item was calculated along with the mean, median, SD, and IQR. Items that satisfied all three criteria were taken into consideration (Agreement 51%, IQR ‹ 1.5, and SD ‹ 1) in the final round. The overall agreement among experts was calculated by Kendall’s coefficient of concordance (Kendall’s W).

## Results

Demographic data

The age group of the panelists was 28-52 years. The panelists were clinicians with 5-20 years of experience. Most of them had a master’s degree (n=21, 70%), followed by a bachelor's degree (n=5, 16.7%), and a few of them had a PhD (n=4, 13.3%). Professions included physiotherapists (n=11, 36.7%), occupational therapists (n=7, 23.3%), ergonomists (n=6, 20%), orthopaedic surgeons (n=4, 13.3%), and physicians (n=2,6.7%) (Table 1).

**Table 1:** Demographic data of the panelists (N=30)

	n (%)	Min	Max	Mean (SD)	Median
Age (years)		28	52	38.3 (7.15)	36.5
Academic qualification					
PhD	4 (13.3)				
Master	21 (70)				
Batchelor	5 (16.7)				
Profession					
Orthopaedic surgeon	4 (13.3)				
Physician	2 (6.7)				
Physiotherapist	11 (36.7)				
Occupational therapist	7 (23.3)				
Ergonomist	6 (20)				
Clinical experience (years)		5	20	10.03 (4.09)	9.5

Round 1

In round one, the data obtained from the literature review and feedback from the participants were compiled together to form the item pool of 41 items. Overall, there were four dimensions (postural alignment, muscular imbalance, functional limitations, and psychosocial factors) in the framework. Four items were included in postural alignment, eight items were included in muscular imbalance, 24 items were included in functional limitation, and five items were included in psychosocial dimensions (Table 2).

**Table 2:** Initial Item pool

Dimensions	Items
Postural alignment	
	Craniovertebral angle
	Kyphotic angle
	Rounded shoulders
	Scapular asymmetry
	Scapular dyskinesia
Muscular imbalance	
	Scapulohumeral rhythm
	Faulty pattern of wall push-up
	Tightness in muscles Pectoralis major, pectoralis minor, Levator scapulae, Sternocleidomastoid
	Deep neck muscles endurance
	Neck muscles strength
	Shoulder muscles strength
	Scapular muscles strength
Functional limitation	
	Cervical range of motion
	Shoulder range of motion
	Thoracic spine mobility
	Neck/Shoulder pain
	Thoracic pain
	Jaw Pain
	Back pain
	Headache
	Trigger point
	Difficulty in household tasks
	Difficulty in jobs
	Difficulty in chin tucks
	Mouth opening
	Fatigue in the neck and arm
	Tightness in the chest
	Peak expiratory flow rate
	Chest expansion
	Dizziness
	Paraesthesia/pins or needle sensation
	Cervical joint position sense
	Static and dynamic balance
	Gait pattern
	Altered arm movement during walking
	Pain in the ribs
Psychosocial factors	
	Poor self-esteem
	Dissatisfaction
	Sleep pattern
	Anxiety and stress
	Social gathering

Round 2

Out of 41 items presented in round one, only 36 items met the criteria selection. Four items in the functional limitation dimension were deleted during this round. The percentage of agreement in this round varied from 46.7% to 96.7%. The overall agreement among the experts was estimated by Kendall’s coefficient of concordance (Kendall's W ranges from zero (no agreement) to one (complete agreement). For round two responses, Kendall’s W was found to be 0.12. The next round of Delphi was conducted due to poor agreement among the experts. The details are provided in Table 3.

**Table 3:** Agreement of the items at the end of Round 2 * Items with agreement IQR: interquartile range

Dimensions	Items	Mean	Median	SD	IQR	% of Agreement
Postural Alignment						
	Craniovertebral angle*	3.80	4	0.61	0	90%
	Kyphotic angle*	3.86	4	0.50	0	93.3%
	Rounded shoulders *	3.66	4	0.75	0	83.3%
	Scapular asymmetry*	3.80	4	0.61	0	90%
Muscular imbalance						
	Scapular dyskinesis*	3.86	4	0.50	0	93.3%
	Scapulohumeral rhythm *	3.93	4	0.36	0	96.7%
	Wall push-up*	3.86	4	0.50	0	93.3%
	Tightness in muscles Pectoralis major, pectoralis minor, levator scapulae, sternocleidomastoid*	3.93	4	0.36	0	96.7%
	Deep neck muscles endurance*	3.60	4	0.81	0	80%
	Neck muscles strength*	3.73	4	0.69	0	86.7%
	Shoulder muscles strength*	3.80	4	0.61	0	90%
	Scapular muscles strength	3.33	4	0.95	2	66.7%
Functional Limitation						
	Cervical range of motion*	3.66	4	0.75	0	83.3%
	Shoulder range of motion*	3.80	4	0.61	0	90%
	Thoracic spine mobility*	3.80	4	0.61	0	90%
	Neck/Shoulder pain*	3.73	4	0.69	0	86.7%
	Thoracic Pain*	3.60	4	0.81	0	80%
	Jaw Pain	3.73	4	0.69	0	86.7%
	Headache*	3.73	4	0.69	0	86.7%
	Trigger point	3.73	4	0.69	0	86.7%
	Difficulty in household tasks*	3.86	4	0.50	0	93.3%
	Difficulty in jobs*	3.73	4	0.69	0	86.7%
	Difficulty in chin tucks*	3.66	4	0.75	0	83.3%
	Mouth opening*	3.73	4	0.69	0	86.7%
	Fatigue in the neck and arm*	3.80	4	0.61	0	90%
	Tightness in the chest*	3.66	4	0.75	0	83.3%
	Peak expiratory flow rate*	3.80	4	0.61	0	90%
	Chest expansion *	3.80	4	0.61	0	90%
	Dizziness*	3.66	4	0.75	0	83.3%
	Back pain	3.06	4	1.01	2	53.3%
	Paraesthesia/pins or needle sensation*	3.73	4	0.69	0	86.7%
	Cervical joint position sense*	3.66	4	0.75	0	83.3%
	Static and dynamic balance*	3.60	4	0.81	0	80%
	Gait pattern	3.06	4	1.01	2	53.3%
	Altered arm movement during walking	3	3	1.01	2	50%
	Pain in the ribs	2.90	2	1.01	2	46.7%
Psychosocial factors						
	Poor self-esteem*	3.73	4	0.69	0	86.7%
	Dissatisfaction*	3.66	4	0.75	0	83.3%
	Sleep pattern*	3.60	4	0.81	0	80%
	Anxiety and stress*	3.66	4	0.75	0	83.3%
	Social gathering*	3.73	4	0.69	0	86.7%

In this round, 37 items were considered. All the items that met the criteria in the second round were maintained in the third round and one item (scapular muscle strength) that had IQR-2 was reconsidered for this round because two out of three criteria were above the threshold. The percentage of agreement in this round varied from 46.7% to 100%. Kendall’s W was found to be 0.31. A moderate level of agreement was reached between the experts and it was decided to conclude the study. The final list was four items in Postural alignment, eight items in Muscular imbalance, 20 items in functional limitation, and five items in Psychosocial factors (Table 4).

**Table 4:** Agreement of the items at the end of Round 3 * Items with agreement; ^ᴓ^ Items without agreement IQR: interquartile range

Dimensions	Items	Mean	Median	SD	IQR	% of Agreement
Postural Alignment						
	Craniovertebral angle*	4	4	0	0	100%
	Kyphotic angle*	4	4	0	0	100%
	Rounded shoulders *	3.80	4	0.61	0	90%
	Scapular asymmetry*	3.93	4	0.36	0	96.7%
Muscular imbalance						
	Scapular dyskinesis*	4	4	0	0	100%
	Scapulohumeral rhythm *	3.80	4	0.61	0	90%
	Wall push-up*	3.86	4	0.50	0	93.3%
	Tightness in muscles Pectoralis major, pectoralis minor, Levator scapulae, Sternocleidomastoid*	4	4	0	0	100%
	Deep neck muscles endurance*	4	4	0	0	100%
	Neck muscles strength*	3.73	4	0.69	0	86.7%
	Shoulder muscles strength*	3.96	4	0.18	0	96.7%
	Scapular muscles strength*	3.80	4	0.61	0	90%
Functional Limitation						
	Cervical range of motion*	4	4	0	0	100%
	Shoulder range of motion*	3.96	4	0.18	0	96.7%
	Thoracic spine mobility*	3.80	4	0.61	0	90%
	Neck/Shoulder pain*	4	4	0	0	100%
	Thoracic Pain*	3.90	4	0.40	0	93.3.%
	Jaw Pain	3.73	4	0.69	0	86.7%
	Headache*	3.85	4	0.50	0	93.3%
	Trigger point	3.96	4	0.18	0	96.7%
	Difficulty in household tasks*	3.90	4	0.40	0	93.3%
	Difficulty in jobs*	3.93	4	0.36	0	96.7%
	Difficulty in chin tucks*	3.85	4	0.50	0	93.3%
	Mouth opening*	3.85	4	0.50	0	93.3%
	Fatigue in the neck and arm*	3.85	4	0.50	0	93.3%
	Tightness in the chest*	3.73	4	0.69	0	86.7%
	Peak expiratory flow rate*	3.93	4	0.36	0	96.7%
	Chest expansion *	3.80	4	0.61	0	90%
	Dizziness*	3.93	4	0.36	0	96.7%
	Back pain ᴓ	3.13	4	1.00	2	56.7%
	Paraesthesia/pins or needle sensation*	3.73	4	0.69	0	86.7%
	Cervical joint position sense*	3.73	4	0.69	0	86.7%
	Static and dynamic balance*	3.86	4	0.50	0	93.3%
	Gait pattern ᴓ	3.13	4	1.00	2	56.7%
	Altered arm movement during walking ᴓ	3.13	4	1.00	2	56.7%
	Pain in the ribs ᴓ	3.06	4	1.01	2	46.7%
Psychosocial factors						
	Poor self-esteem*	3.80	4	0.61	0	90%
	Dissatisfaction*	3.73	4	0.69	0	86.7%
	Sleep pattern*	3.73	4	0.69	0	86.7%
	Anxiety & stress*	3.80	4	0.61	0	90%
	Social gathering*	3.93	4	0.36	0	96.7%

## Discussion

The objective of the study was to develop a conceptual framework for UCS. This study will provide a better understanding of UCS and the areas that have to be emphasized for the correction and rehabilitation of this syndrome.

The first dimension identified is postural alignment. A previous study by Seidi et al. mentioned that this syndrome can lead to an imbalance in the postural alignment [7]. The second dimension found was muscular imbalance. Janda has already suggested that there is a pattern of muscular imbalance in UCS due to the tightness of the upper trapezius, pectorals, and levetor scapulae and weakness of the middle and lower fibers of the trapezius, infraspinatus, and serratus anterior [1]. This muscular imbalance can lead to altered arthrokinematics and movement patterns [2]. Muscle imbalance can have an impact on movement patterns. Faulty movement patterns may lead to structural changes; therefore, thorough knowledge will help us to guide various prevention strategies.

Good posture is important for optimal functional performance [15]. Muscular imbalance can lead to limitations in the functions. Moreover, this study is in line with the International Classification of Functioning, Disability, and Health (ICF) model of disability and functioning. The multidimensional model emphasizes that activity limitation may occur due to problems in the body or structure [16]. This can lead to restriction in the range of motion of neck or shoulder joints [15] and many other secondary complications if not treated.

The healthcare model must be psychosocial. The individual and social contexts of the person's experience of health were taken into consideration by the ICF model, in addition to the biological aspect of health [11]. Good posture can impact one's sense of self-worth, which in turn can impact one's social relationships and personal life [12]. Individuals with proper posture will not only feel more confident, but their psychosocial status will also improve.

This conceptual framework will provide a broader horizon for further research. A multidimensional approach of corrective strategies will help in the early rehabilitation of UCS. Educating the patient to maintain good postural habits is important to prevent this syndrome. A thorough knowledge of the concept will provide a better understanding of the prevention strategies that will prevent the syndrome from progressing and further structural changes can be avoided.

The strength of the current study was that 30 multidisciplinary experts were chosen with clinical experience varying from five to 20 years. Their valuable insights helped to reach a consensus after three Delphi rounds. In this study, we used the Delphi method, which is a cost-effective method with small to medium sample size in contrast to other research methods that require a medium to large sample size [17]. Besides this, Delphi is a flexible method of electronic communication between researchers and experts which makes it an ideal approach to reach consensus [18]. Moreover, the innovative nature of the study adds to the existing knowledge gap.

One of the limitations of the study was that all the experts were Indian in origin, so it is difficult to draw conclusions on a global basis. Secondly, the lack of defined criteria for expert selection was another limitation of this study. Lastly, only experts’ opinions (clinicians) were taken into consideration, but inputs from the patients with UCS would have added more to this study. Integration of a patient-centered approach in the healthcare delivery system improves patient engagement, satisfaction, and quality of life [19]. Future studies can be done by including all the stakeholders, especially the patient population, who have to face this problem on a daily basis.

## Conclusions

We successfully developed a conceptual framework for UCS. This framework will help to understand various aspects of UCS and its impact that requires evaluation at different stages of progression. In clinical settings, this will help clinicians to identify UCS and provide treatment strategies for both prevention and cure. The multidimensional approach of the study will provide a better scope for clinicians to educate patients about good posture, which not only will impact physical performance but also improve the quality of life. The development of this framework will help to prevent, monitor the progress, and correct UCS. This study will form a baseline and open doors for future research. Future studies can be done by including experts and patient populations from different countries that will help to study the cross-cultural and geographical differences and validate the framework on a global basis. 
